# Co-occurrence of Rapid Gene Gain and Loss in an Interhospital Outbreak of Carbapenem-Resistant Hypervirulent ST11-K64 *Klebsiella pneumoniae*

**DOI:** 10.3389/fmicb.2020.579618

**Published:** 2020-11-12

**Authors:** XiaoTuan Zhang, JingLin Ouyang, WenWen He, Tong Zeng, Bin Liu, Hongtao Jiang, Yunsheng Zhang, Linlin Zhou, Haijian Zhou, Zhuoran Liu, Logen Liu

**Affiliations:** ^1^Clinical Laboratory, The Second Affiliated Hospital, University of South China, Hengyang, China; ^2^Department of Ultrasound Medicine, The Second Affiliated Hospital, University of South China, Hengyang, China; ^3^Department of Organ Transplantation, The Second Affiliated Hospital, Hainan Medical University, Haikou, China; ^4^Clinical Research Center, The Second Affiliated Hospital, University of South China, Hengyang, China; ^5^State Key Laboratory for Infectious Disease Prevention and Control, National Institute for Communicable Disease Control and Prevention, Chinese Center for Disease Control and Prevention, Beijing, China

**Keywords:** *Klebsiella pneumoniae*, antibiotic resistance, virulence factors, microevolution, mobile genetic elements

## Abstract

We report an outbreak of carbapenemase-producing hypervirulent *Klebsiella pneumoniae* in two hospitals that undergo frequent patient transfers. Analysis of 11 completely assembled genomes showed that the bacteria were ST11-K64 strains. Moreover, 12 single nucleotide polymorphisms (SNPs) identified the strains as having originated from the same cluster, and were also indicative of the interhospital transmission of infection. Five plasmids were assembled in each of the strains. One plasmid carried several virulence genes, including the capsular polysaccharide regulators *rmpA* and *rmpA2*. Two others carried antimicrobial-resistance genes, including one for carbapenem resistance, *bla*_KPC–2_. Comparative genomic analysis indicated the occurrence of frequent and rapid gain and loss of genomic content along transmissions and the co-existence of progeny strains in the same ward. A 10-kbp fragment harboring antimicrobial resistance-conferring genes flanked by insert sequences was missing in a plasmid from strain KP20194c in patient 3, and this strain also likely subsequently infected patient 4. However, strains containing the 10-kbp fragment were also isolated from the ward environment at approximately the same time, and harbored different chromosome indels. Tn*1721* and multiple additional insert sequence-mediated transpositions were also seen. These results indicated that there is a rapid reshaping and diversification of the genomic pool of *K. pneumoniae* facilitated by mobile genetic elements, even a short time after outbreak onset. ST11-K64 CR-hvKP strains have the potential to become new significant superbugs and a threat to public health.

## Introduction

*Klebsiella pneumoniae* is a gram-negative bacterium that can cause both community-acquired and nosocomial infections. Carbapenem-resistant *K. pneumoniae* (CRKP) strains pose a substantial threat to public health. Four major types of carbapenemases have been identified globally: *bla*_KPC–like_, *bla*_OXA–48–like_, *bla*_NDM–like_, and *bla*_VIM–like_ (Brink, 2019; Cui et al., 2019). The *bla*_OXA–48_ gene was first reported in Turkey and is mainly concentrated in Middle Eastern and European countries (Poirel et al., 2004). *bla*_NDM_ was first identified in India, but has since disseminated throughout South Asia, and now represents the second leading cause of carbapenem resistance in China (Zhang et al., 2017). Greece is the epicenter of *bla*_VIM_ (Matsumura et al., 2018), while *bla*_KPC_ is widespread in the United States, South America, and China, with *bla*_KPC–2_ being the most frequently identified carbapenemase in the latter country (Zhang et al., 2018). Most CRKP strains distributed in China are ST11, while its descendent strain, ST258, which arose from a recombinant event between ST11 and ST442, is dominant in the United States and other western countries (Chen et al., 2014; Zhang et al., 2017).

The hallmark clinical manifestation of hypervirulent *K. pneumoniae* (hvKP) infection is a hepatic abscess (Liu et al., 1986). Young and healthy individuals are also vulnerable to hvKP, with diabetes as a risk factor. A hypermucoviscous phenotype due to increased *rmpA* and/or *rmpA2*-regulated capsule expression and enhanced iron acquisition through aerobactin, salmochelin, yersiniabactin, and enterobactin are characteristics of hvKP; however, no single specific molecular marker has been identified (Russo and Marr, 2019). The first reported hvKP cases were sensitive to antimicrobial reagents, and mostly belonged to K1 and K2 capsular types (Russo and Marr, 2019). However, recently reported cases or outbreaks of *K. pneumoniae* infection showed convergent virulence and resistance, resulting in the emergence of CR-hvKP, and the K47 and K64 serotypes, in addition to K1 and K2 (Zhang et al., 2016; Feng et al., 2018; Gu et al., 2018; Shen et al., 2019; Zhao et al., 2019). The mortality rate associated with these infections is extremely high, especially in immunocompromised patients in intensive care units (ICU).

*Klebsiella pneumoniae* exhibits rapid genomic evolution, which increases the likelihood of the emergence of new virulence and resistance-conferring genes. A comparison of the complete genome of three CR-hvKP strains spanning approximately 5 months revealed the presence of three major indels that confer colistin resistance (Dong et al., 2018; Gu et al., 2018). Here, we report an outbreak and transmission of CR-hvKP in two hospitals in a province of central south China, the characterization of its molecular basis, and a comparison of the complete genomic content of 11 strains. Our results confirmed that *K. pneumoniae* has a rapidly evolving genome and revealed the co-existence of differentially evolved strains in one ward. ST11-K64 CR-hvKP strains have the potential to become one of the dominant *K. pneumoniae* serotypes in China in the next few years alongside the hypervirulent K1 and K2.

## Materials and Methods

### Bacterial Strains

From March to May 2019, a total of 13 CR-hvKP isolates were collected from patients or the surfaces of ward equipment in two hospitals. The CR-hvKP outbreak occurred between secondary and tertiary hospitals. An antibiotic susceptibility test (AST), carbapenemase gene identification, and whole-genome sequencing (WGS) analysis were performed for each isolate. The observation endpoint was defined as a discharge or transfer from the hospital. The research was approved by the Medical Research Ethics Committee of The Second Affiliated Hospital, University of South China.

### ASTs and String Test

Antibiotic susceptibility tests were performed for the 13 isolates using the VITEK 2 Compact system (bioMérieux, Marcy l’Etoile, France), an Epsilometer test (E-test), or the disk-diffusion method, and the results of the ASTs were interpreted as recommended by the Clinical and Laboratory Standards Institute, version 2019 (CLSI, 2019). Tests were performed for tigecycline, polymyxin B, and ceftazidime/avibactam in addition to regular monitoring drugs. *K. pneumoniae* colonies grown on blood agar plates overnight were stretched with a loop, and a positive string test was defined as the formation of viscous strings greater than 5 mm in length.

### Genomic DNA Extraction, Sequencing, and Genome Assembly and Annotation

Genomic DNA was extracted from log-phase *K. pneumoniae* using a genomic DNA extraction kit (Thermo GenJet, United States). In total, 400 ng of DNA from each sample was fragmented and barcoded using a Rapid Barcoding Sequencing Kit (Oxford Nanopore, United Kingdom), and then mixed and purified with AMPure XP beads (Beckman, United States). The purified mix was then loaded into a flow cell (R9.4.1) and sequenced using a MinION sequencer (Oxford Nanopore). Base-calling, quality control, and demultiplexing were performed with the Guppy software. Library construction and shotgun sequencing in the Illumina platform were performed by Novogene (Beijing, China). SPAdes was used for the *de novo* assembly of short Illumina reads (Bankevich et al., 2012). The Unicycler pipeline (Wick et al., 2017), which integrates several software programs for the hybrid assembly, polish, and circularization of long and short reads, was used to obtain the final genome sequence.

### Sequence Types, Antimicrobial Resistance Genes, Virulence Genes, and Plasmids

K-locus (polysaccharide capsule) typing was identified with the Kaptive software (Wick et al., 2018), with whole-genome or assembled scaffold sequences. Multilocus sequence typing and the identification of antimicrobial resistance-conferring genes were performed using the MLST 2.0 and ResFinder 3.2 webservers (Thomsen et al., 2016) at the Center for Genomic Epidemiology (CGE). Virulence genes were identified in the Virulence Factors Database (VFDB) with Rapid Annotation using Subsystem Technology (RAST)-annotated GenBank files (Overbeek et al., 2014; Liu et al., 2019). Incompatibility groups of plasmids were identified through the PlasmidFinder 2.0 webserver (Thomsen et al., 2016) at CGE. Circular structure maps were drawn using the Brig software (Alikhan et al., 2011).

### Single Nucleotide Polymorphisms (SNPs), Deletions/Insertions, and Phylogenetic Analysis

Pangenomic SNPs across the finished genomes of the 11 strains were found using kSNP3.1 (Gardner et al., 2015) with the “-core” parameter specified, which only showed the SNPs present in all 11 strains. The identified SNPs were then manually checked and annotated. Global genome alignment was performed with Mauve. The regions harboring potential large insertion and deletion (indel) fragments, as revealed by Mauve, were further extracted and aligned using EMBOSS Stretcher, manually checked for detailed indel sites, and then annotated. The PCR primers used for the verification of the indels of interest are listed in Supplementary Table 1. Core genome SNP (cgSNP) and core genome MLST (cgMLST) phylogenetic analyses were performed in the BacWGSTdb (Ruan and Feng, 2016) webserver (accessed on May 20, 2020). The genome sequence of strain KP20194a was first uploaded to the webserver for “single genome analysis.” The cgMLST phylogenetic tree was generated for related strains (differences in ≤50 loci), and strains harboring differences in less than 200 SNPs were retrieved. The retrieved genomes, together with the 11 strains in this study (listed in Supplementary Table 2), were then again uploaded to BacWGSTdb for “multiple genome analysis” of the phylogenetic relationship based on a SNP strategy.

## Results

### Tracking of Outbreak Interhospital Transmission

**FIGURE 1:**
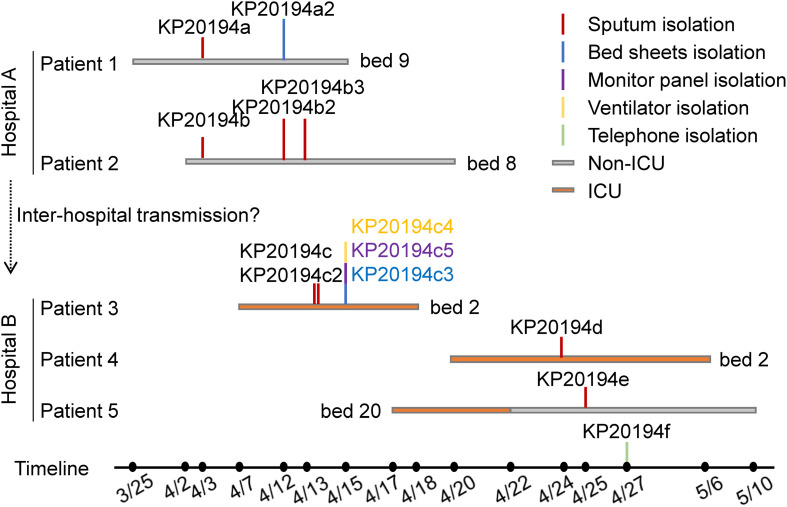
Chronological order of the sampling of the sequenced CR-hvKP isolates. Each horizontal line represents the admission and discharge date of a patient, and the vertical lines indicate the day and location of the collection of the *K. pneumoniae* strains. The environmental samples were as follows: KP20194c3, the bedsheets of patient 3; KP20194c4, ventilator; KP20194c5, monitor panel; and KP20194f, the telephone of the nurse station in the intensive care unit (ICU). Patients 1 and 2 were from hospital A; patients 3, 4, and 5 were from hospital B.

Our 13 isolates were cultured from samples obtained from five patients and from the environment during an outbreak of multidrug-resistant *K. pneumoniae* in two hospitals that had frequent interhospital patient transfer. Between late March and April 2019, four CR-hvKP strains were isolated from the sputum of two patients presenting with critical lung infections at the neurosurgery unit of a secondary hospital (hospital A, Figure 1), and one strain was isolated from the bedsheets of patient 1. Shortly after, four CR-hvKP strains were isolated from the sputum of three patients and four from the environment at the integrated ICU of a tertiary hospital (hospital B, Figure 1). Notably, patients and medical staff were routinely exchanged between the two hospitals. All the isolates were hypermucoviscous as judged by positive string tests; however, no liver abscesses or bloodstream infections were detected. Patients 1 and 2 were admitted to the same ward of hospital A and occupied adjacent beds. Patient 4 used the same bed and ventilator as patient 3 in the ICU of hospital B after the latter was discharged. Patient 5 was transferred to another surgery ward after a 3-day stay in the ICU.

### Complete Genome Analysis Revealed a Single ST11-K64 Cluster

Strains KP20194b2 and KP20194b3 were both isolated from patient 2 on April 12 and April 13, while strains KP20194c and KP20194c2 were isolated from patient 3 on April 13. Given that strains isolated by repeated sampling within a 1-day interval may present no significant changes in molecular characteristics, strains KP20194b2 and KP20194c, as well as the remaining 9 strains (11 in total), were selected for further Oxford Nanopore sequencing to obtain complete genome sequences. A circularized 5.4 Mbp chromosome was obtained from all the 11 strains, all of which also contained five additional plasmids (Supplementary Table 2). The size of the chromosomes ranged from 5447573 bp to 5450212 bp. Multilocus sequence typing showed that they belonged to ST11 *K. pneumoniae* strains, which are prevalent in China and south-eastern Asia, and have been linked to carbapenem resistance (Zhang et al., 2017). The K-locus type was identified as K64, which is relatively “less common” among KPC2 carbapenemase-producing strains in China.

### Loss of a 10-kbp Fragment Resulted in Two Resistant Profiles

**FIGURE 2:**
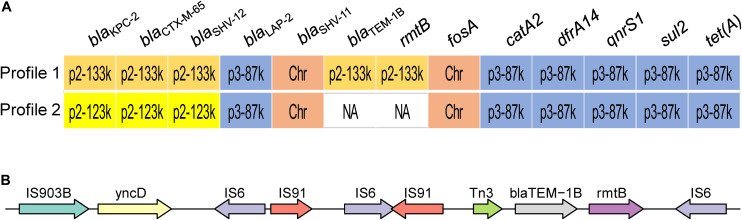
Outbreak isolates present two antibiotic susceptibility profiles. **(A)** The antimicrobial resistance-conferring genes of the two profiles. The location of the resistance genes in the chromosome (Chr) or plasmids p2 and p3 (followed by the size of the respective plasmid) is indicated. Profile 1: strains KP20194a, KP20194a2, KP20194b, KP20194b2, KP20194b3, KP20194c3, KP20194c4, KP20194e, and KP20194f. Profile 2: strains KP20194c, KP20194c2, KP20194c5, and KP20194d. **(B)** A representative of the content of the lost 10-kbp fragment from profile 2 that resulted in the sensitivity to amikacin.

Two profiles of drug susceptibilities could be observed within the 13 strains, i.e., resistant or susceptible to aminoglycoside (Figure 2A). All the isolates were resistant to the beta-lactams tested, including meropenem and imipenem (Table 2). Screening using the CGE webserver revealed that the antibiotic resistance of these strains was mainly endowed by two plasmids: plasmid p2 (123 or 133 kbp) carried resistance genes for beta-lactams and aminoglycosides, while plasmid p3 (89 kbp) carried resistance genes to phenicols, (fluoro)quinolones, rifampicin, sulfonamide, tetracycline, and trimethoprim (Figure 2A). An additional chromosome-borne beta-lactamase gene *bla*_SHV–11_ was also found, which is common in *K. pneumoniae* (Lee et al., 2006). Owing to its high similarity with the plasmid-borne *bla*_SHV–12_, it could only be discriminated by long-read sequencing. Carbapenem resistance was conferred by *bla*_KPC–2_ located in plasmid p2. Interestingly, *bla*_TEM–1B_ and *rmtB*, located in the resistance island of plasmid p2, were lost in latter strains (Profile 2, strains KP20194c, KP20194c2, KP20194c5, and KP20194d), resulting in a plasmid of 123 kbp. As shown in Figure 2, further analysis of this lost 10-kbp fragment in the plasmids (pKP20194c-p2, pKP20194c2-p2, pKP20194c5-p2, and pKP20194d-p2) of profile 2 strains showed that *bla*_TEM–1B_ and *rmtB* were surrounded by sets of IS6 and IS903B insertion sequences. The loss of *rmtB* resulted in susceptibility to aminoglycosides, as evidenced by the amikacin and gentamicin antibiotic susceptibility test (Table 2). The loss of *rmtB* was also confirmed by PCR (Supplementary Figure 1). Strains KP20194c4 and KP20194c5 were collected on the same day; strains KP20194d, KP20194e, and KP20194f were collected at approximately the same time from different patients or ward surfaces. However, KP20194c5 and KP20194d harbored the 123 kbp plasmid, while KP20194f, KP20194c4, and KP20194e harbored the 133 kbp plasmid, suggesting that these progeny strains co-existed in the same ward at around the same time.

### Virulence Gene Analysis Revealed CR-hvKP Convergence by Virulence Plasmid Picking-Up

**FIGURE 3:**
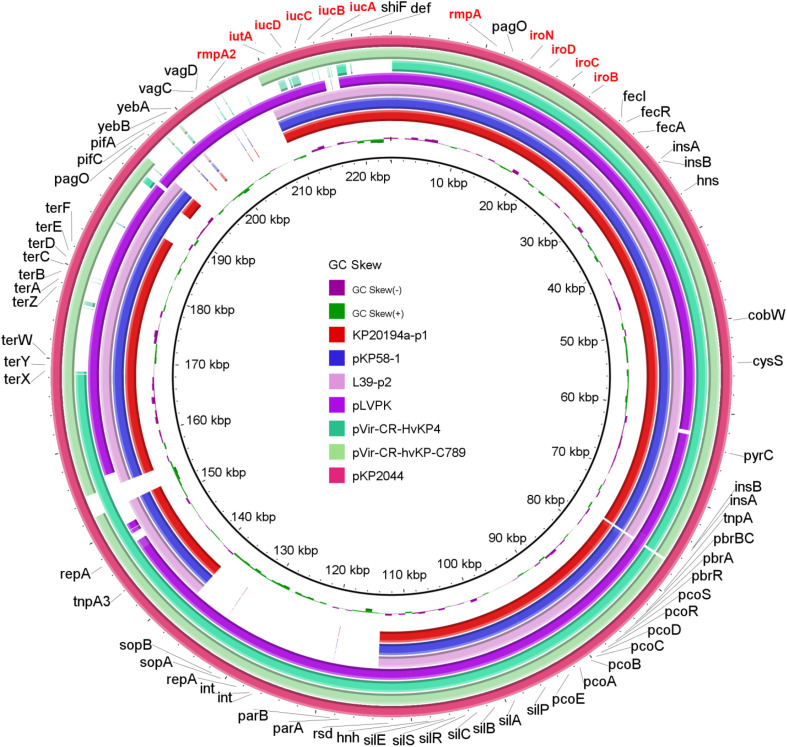
Genetic comparison of the virulence plasmid. BRIG comparison of seven plasmids, including the classical virulence plasmids pLVPK and pKP2044; pVir-CR-hvKP4 from an outbreak of the ST11-K47 strain (Gu et al., 2018); pKP58-1 from a recently reported single ST11-K64 strain (Ruan et al., 2020); pVir-CR-hvKP-C789 from the ST11-K64 strain reported in a retrospective analysis that indicated the early spread of these strains (Zhang et al., 2020); and an L39-p2 strain that exhibited a high coverage and identity with plasmid p1 from our KP20194a strain (by BLAST). pKP2044 was used as the reference plasmid. Virulence genes are highlighted in red.

A hypermucoviscous phenotype is believed to be one of the characteristics of hvKP. We performed a string test, and all 13 strains tested produced viscous strings of >5 mm. *rmpA* and *rmpA2*, both of which were reported to enhance capsular polysaccharide production (Russo and Marr, 2019), were detected in these 13 strains. Moreover, these two genes were located in plasmid p1 (195 kbp, Figure 3) in the 11 strains with sequenced genomes. hvKP contains four types of siderophore clusters for iron acquisition. In our 11 strains with sequenced genomes, aerobactin (*iucABCD* and *iutA*) and salmochelin (*iroBCDN*) were located on plasmid p1, while enterobactin (*entABCDEFS*, *fepABCDG*, and *fes*), salmochelin (*iroEN*), and yersiniabactin (*ybtAEPQSTUX*, *irp1/2*, and *fyuA*) were located on the chromosome. This pattern of virulence gene distribution is very similar to that of the classical hypervirulent strain NTUH-K2044 (Wu et al., 2009). A BLAST comparison of the virulence genes present in plasmid p1 with those of highly similar counterparts identified in NCBI GenBank showed high similarity between plasmid p1 and pKP58-1 (197 kbp, 99% coverage, and 100% identity) and L39-p2 (198 kbp, 99% coverage, and 99.97% identity). These three plasmids are smaller than the classical hypervirulent plasmids pKP2044 and pLVPK (Figure 3).

### Phylogenetic Analysis of Closely Related Strains

cgSNP and cgMLST analyses were performed to evaluate the phylogenetic relationship between our strains and related ST11 strains. Twelve SNPs were found among the outbreak strains, and their detailed information for the 11 strains with complete genome sequence is shown in Supplementary Table 3. Three SNPs resulted in amino acid changes in protein-coding genes, including a change in a hypothetical protein. Three mutations were found in the gene coding for L-threonate dehydrogenase (*ItnD*; a hotspot for mutation), one of which was a valine to alanine substitution at position 162 (V162A). A synonymous SNP was found in *bla*_SHV–11_. These SNP results confirmed that these strains were from the same outbreak cluster, aligning to the criteria suggested by the EuSCAPE Working Group that 21 is the optimal number of SNPs to discriminate ST258 hospital clusters (David et al., 2019). cgMLST analysis revealed four locus differences among the outbreak strains. Three of them (KP1_RS00760, KP1_RS04975, KP1_RS08550) could not been assigned to the genome assembly of strain KP20194c3. And locus KP1_RS08610 could not been assigned to the genome assemblies of both KP20194b and KP20194b2. These results further confirmed that they belonged to the same cluster.

**FIGURE 4:**
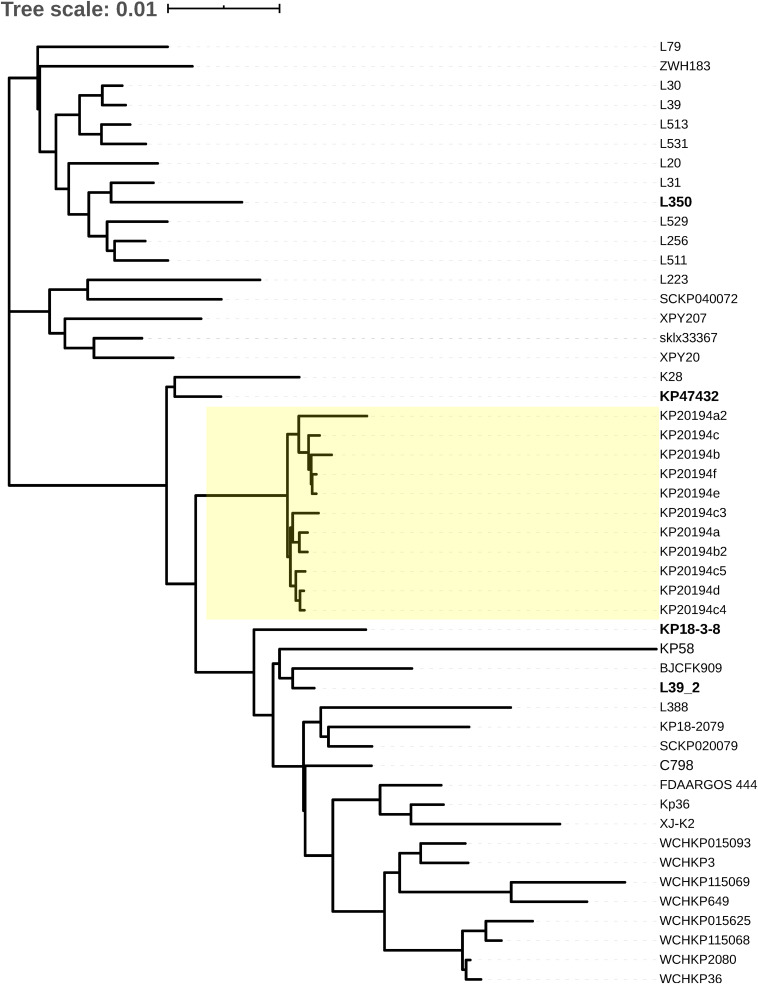
Core genome SNP phylogenetic analysis of our outbreak strains compared with closely related strains. The outbreak strains in this study are highlighted in light yellow. The related strains mentioned in the main text are indicated.

The cgSNP and cgMLST alleles differing between the closely related strains and our strains (KP20194a as reference strain), together with their collection location and date, are listed in Supplementary Table 4. Most of the closely related strains were collected in China in the last 5 years, except strain FDAARGOS_444, which was collected in Canada in 2013. The phylogenetic trees constructed based on cgSNP and cgMLST are shown in Figure 4 and Supplementary Figure 2, respectively. The outbreak strains in this study clearly clustered into one clade based on cgSNP (Figure 4). The related strains included KP47432 and KP18-3-8, two ST11-K64 strains collected from bloodstream infection and a urine sample, and showed 66 and 88 SNPs differences, respectively, compared with strain KP20194a (Figure 4 and Supplementary Table 4). Strain L350 (acc. NLDZ01) showed 13 cgMLST loci differences compared with strain KP20194a (Supplementary Figure 2 and Supplementary Table 4). Strain L39_2, which shared the most similar plasmid content with our strains, differed in 117 cgSNP and 15 cgMLST loci. Strain L350 and L39_2 are both ST11-K64 strains collected from the stool of patients with acute diarrhea (Zheng et al., 2020). Three of these strains (KP47432, L350, and L39_2) were collected in Hangzhou, China.

### The Co-existence of Major Indels

**FIGURE 5:**
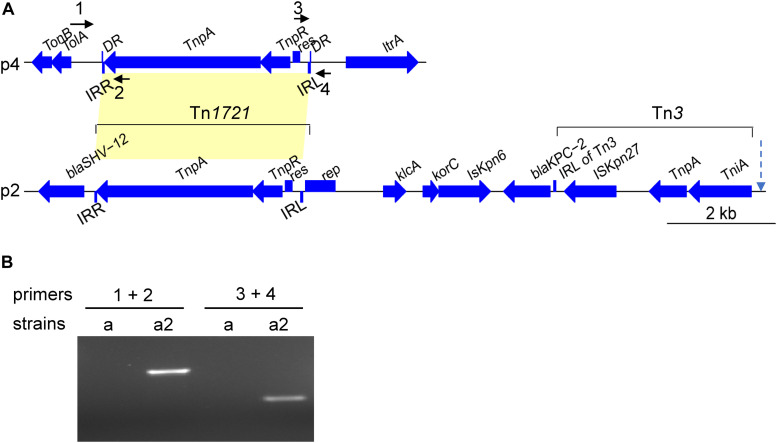
Schematic of a Tn*1721*-mediated plasmid-to-plasmid translocation. **(A)** The genetic content of Tn*1721* translocated to plasmid p4 and the original KPC locus carried by Tn*1721* from plasmid p2 in strain KP20194a2. IRL, the left terminal inverted repeat of Tn*1721*; IRR, the right terminal inverted repeat of Tn*1721*. DR, Tn*1721*-mediated translocation resulted in the generation of a 5-bp direct repeat at the target site. Arrow with a dotted line indicates the site of the additional IRL2 in Tn*1721*-like transposon-mediated *bla*_KPC–2_ translocation reported elsewhere (Tang et al., 2017), which was not seen in our strains or other highly similar plasmids from strains KP58 and L39_2. The arrows around Tn*1721* indicate the relative location of the designed primers (but not the length). **(B)** PCR was performed with the indicated primers using genomic DNA extracted from KP20194a or KP20194a2 as a template to confirm the targeted insertion. The amplified fragment was verified by Sanger sequencing. Strains: a, KP20194a; a2, KP20194a2. Primer locations are indicated in **(A)** and sequences in Supplementary Table 1.

We compared the major plasmid- and chromosome-borne indels among the 11 strains with the sequenced genomes. Besides the above-mentioned loss of the 10-kbp fragment in plasmid p2 of KP20194c, several distinct genomic indels were observed. First, a 4-kbp Tn*1721* fragment, which was originally located in plasmid p2 (133 kbp), was “copied and pasted” into plasmid p4 of strain KP20194b2, resulting in a plasmid of approximately 15 kbp (Figure 5A). *Tn*3-based transposons (Tn*4401* in western countries and the Tn*1721*-like transposon in China) were important for the dissemination of the *bla*_KPC–2_ gene (Cuzon et al., 2011; Tang et al., 2017). Although *bla*_KPC–2_ was not translocated in this study, possibly owing to the lack of an additional left terminal inverted repeat (IRL2) of Tn*1721* after Tn*3* in plasmid p2 (Figure 5A). To confirm the insertion, which was based on Unicycler software assembly, we performed PCR using primers designed around the insert sites. A fragment with the predicted size was amplified from the genomic DNA of strain KP20194b2, but not from its parent strain KP20194b (Figure 5B), which had also been isolated from patient 2 nine days previously. Sanger sequencing confirmed the “copied” fragment and a “TATAC” pentanucleotide directed repeat (DR) at the insert site. Several other indels were also identified (Table 2), including some mediated by *InsH* and IS3. Strains sampled during a 2-month period have been reported to harbor different indels (Dong et al., 2018). However, our results revealed that strains sampled within approximately 10 days from different sources in the same ward also harbored different indels, indicative not only of a rapidly evolving genome mediated by mobile genetic elements but also the co-existence of progeny strains.

## Discussion

Here, we report an interhospital outbreak of CR-hvKP. The outbreak was first identified in hospital A, where patients 1 and 2 were infected. Although they were not transferred to hospital B, three other patients were infected in the ICU and neural surgical ward of the latter hospital (B). The detailed transmission route was not traceable because of the frequent transfer of patients between these two hospitals and the frequent co-operation of medical staff. However, molecular tracking clearly indicated that they formed part of the same outbreak cluster, as suggested by the limited cgSNP and cgMLST and whole-genome comparisons (Tables 1, 2). Strain KP20194d, isolated from patient 4, and strain KP20194c, isolated from patient 3, shared the loss of a 10-kbp fragment from plasmid p2 which contained *rmtB* (Figures 1, 2), suggesting the transmission from patient 3 to patient 4 given the timeline and the use of the same bed and ventilator.

**TABLE 1:** Resistance profiles of the 13 strains of the outbreak.

Profile	IMP	MEM	SCF	TZP	TIM	FOX	FEP	CAZ	CRO	CIP	GEN	AMK	PB	TGC	CZA
1	≥16	≥16	≥64/32	≥128/4	≥128/2	≥32	≥32	≥32	≥64	≥4	≥16	≥64	1	0.5	23 mm (S)
2	≥16	≥16	≥64/32	≥128/4	≥128/2	≥32	≥32	≥32	≥64	≥4	**≤**1	**≤**4	1	0.5	23 mm (S)

*IMP, imipenem; MEM, meropenem; SCF, cefoperazone/sulbactam; TZP, piperacillin/tazobactam; TIM, ticarcillin/clavulanic acid; FOX, cefoxitin; FEP, cefepime; CAZ, ceftazidime; CRO, ceftriaxone; CIP, ciprofloxacin; CXM, cefuroxime; GEN, gentamicin; AMK, amikacin; PB, polymyxin B; TGC, tigecycline; CZA, ceftazidime/avibactam, S, sensitive (≥21 mm). Profile 1, strains KP20194a, KP20194a2, KP20194b, KP20194b2, KP20194b3, KP20194c3, KP20194c4, KP20194e, and KP20194f. Profile 2, strains KP20194c, KP20194c2, KP20194c5, and KP20194d.*

**TABLE 2:** Major indels in the chromosome of the outbreak strains.

Strain	Reference strain	Start site of reference strain	Start site of query strain	Indel	Length	Content
KP20194a2	KP20194a	3,015,188	3,015,188	D	1,197	Hypo
		3,456,349	3,355,153	I	268	Hypo
KP20194b2	KP20194b	3,015,028	3,015,028	I	598	Hypo
		4,506,947	4,507,546	I	1,474	IS3
KP20194c3	KP20194c	4,039,989	4,039,989	I	1,200	InsH
KP20194c4	KP20194c	3,456,264	3,456,532	D	183	–
		4,039,989	4,039,806	I	1,200	InsH
KP20194c5	KP20194c	3,459,773	3,459,772	I	182	–
KP20194f	KP20194a	3,456,349	3,456,350	I	182	–

*Hypo, hypothetical protein; D, deletion; I, insertion.*

The emergence of the CR-hvKP strains can result from the acquisition of either a carbapenemase-producing plasmid by a hypervirulent strain, usually belonging to serotype ST23 and capsular type K1/K2 (Yao et al., 2015; Dong et al., 2019; Shen et al., 2019); or a pLVKP-like virulence plasmid by CRKP strains, among which ST11 is dominant in China (Wei et al., 2016; Ruan et al., 2020; Yang et al., 2020). Our strains likely fit into the latter category, because MLST and Kaptive software analyses indicated that our 13 strains belonged to ST11-K64, and also because of the existence of the pLVPK-like virulence plasmid (Figure 3). The capsular type of K64 has not frequently been reported so far. *K. pneumoniae* with K47 and K64 capsule types may be emerging as the dominant strains in China. When the landmark convergence of CR-hvPK was originally reported, the strains referred to were K47 (Gu et al., 2018). Moreover, when Yang et al. (2020) reported the emergence of ST11-K47/K64 CR-hvKP, the authors stated that ST11-K64 was relatively rare. However, a subsequent retrospective multicenter study showed that ST11-K64 must have been spreading in China for several years and represents the most common type of CR-hvKP (Zhang et al., 2020). Indeed, de Campos et al. (2018) reported a hypermucoviscous CRKP strain that caused a fatal bacteremia in a patient in Brazil in 2018. Our report of an interhospital outbreak of ST11-K64 provided further evidence that ST11-K64 *K. pneumoniae* may be a competent host strain for a hypervirulent plasmid, leading to CR-hvKP, and control measures should be urgently implemented.

In summary, this study revealed the molecular epidemiology of an interhospital outbreak of CR-hvKP and tracked the microevolutionary events. We highlighted the rapid evolution of the CR-hvKP genome and the co-existence of CR-hvKP progeny strains. The emerging and increasingly reported ST11-K64 CR-hvKP strains require urgent control measures.

## Data Availability Statement

The complete whole-chromosome (genome) and plasmid sequences of the 11 strains have been deposited in GenBank with accession numbers from cp054720 to cp054785.

## Ethics Statement

The studies involving human participants were reviewed and approved by Medical Ethics Committee, The Second Affiliated Hospital, University of South China. The ethics committee waived the requirement of written informed consent for participation.

## Author Contributions

LL, ZL, HZ, and XZ designed the project. XZ, JO, WH, TZ, LZ, HZ, and LL performed the experiments. XZ, BL, HJ, YZ, and LL analyzed the data. XZ, LZ, and LL wrote the manuscript. All authors read and approved the final manuscript.

## Conflict of Interest

The authors declare that the research was conducted in the absence of any commercial or financial relationships that could be construed as a potential conflict of interest.
