# Supplementary material for: Co-occurrence of Rapid Gene Gain and Loss in an Interhospital Outbreak of Carbapenem-Resistant Hypervirulent ST11-K64 Klebsiella pneumoniae
Source: Front Microbiol. 2020 Nov 12;11:579618. doi: 10.3389/fmicb.2020.579618 (PMC7688671; doi:10.3389/fmicb.2020.579618)
Supplement: Supplementary file 1 [file Data_Sheet_1.DOCX]

Supplementary Material

# Supplementary Figures

**Supplementary Figure 1.** PCR of *rmtB* to verify the lost fragment in strains from resistance profile 2. Genomic DNA from the indicated strains was PCR amplified with primers rmtB-F and rmtB-R (Supplementary Table 1) and separated by agarose gel electrophoresis. No band corresponding to *rmtB* was amplified from the genomic DNA of strains from resistance profile 2.

**Supplementary Figure 2.** The phylogenetic relationship between *Klebsiella* *pneumoniae* KP20194a and closely related strains based on cgMLST. Phylogenetic grape tree of the strains retrieved from BacWGSTdb exhibiting less than 50 cgMLST loci differences compared with KP20194a. The circles with different color indicate strains from different regions of China.

# Supplementary Tables

Supplementary Table 1. Primers used in this study

| Primer_name | Sequence |
| --- | --- |
| rmtB-F | acttttacaatccctcaatac |
| rmtB-R | aagtatataagttctgttccg |
| Primer 1 | gtggtttccgtagcctcg |
| Primer 2 | gccagctcgatcaagcag |
| Primer 3 | tcgtttccatgctagctt |
| Primer 4 | gtatgcaacgcatgactc |

Supplementary Table 2. Complete genome size of the 11 strains (bp)

| Patient ID | Isolation date | Strain | Chr | p1 | p2 | p3 | p4 | p5 | Total |
| --- | --- | --- | --- | --- | --- | --- | --- | --- | --- |
| 1 | 2019/04/03 | KP20194a | 5448829 | 195031 | 133771 | 87095 | 11970 | 5596 | 5882292 |
| 1 | 2019/04/12 | KP20194a2 | 5447900 | 195030 | 133772 | 87095 | 15931 | 5596 | 5885324 |
| 2 | 2019/04/03 | KP20194b | 5447537 | 195034 | 133772 | 87095 | 11970 | 5596 | 5881004 |
| 2 | 2019/04/12 | KP20194b2 | 5449609 | 195077 | 133772 | 87095 | 11970 | 5596 | 5883119 |
| 3 | 2019/04/13 | KP20194c | 5449012 | 195024 | 123177 | 87095 | 11970 | 5596 | 5871874 |
| 3 | 2019/04/15 | KP20194c3 | 5450212 | 195028 | 133772 | 87095 | 11970 | 5596 | 5883673 |
| 3 | 2019/04/15 | KP20194c4 | 5450029 | 195025 | 133772 | 87095 | 11971 | 5596 | 5883488 |
| 3 | 2019/04/15 | KP20194c5 | 5449193 | 195034 | 123177 | 87095 | 11970 | 5596 | 5872065 |
| 4 | 2019/04/24 | KP20194d | 5448829 | 194896 | 123177 | 87095 | 11970 | 5596 | 5871563 |
| 5 | 2019/04/25 | KP20194e | 5448829 | 195027 | 133772 | 87095 | 11970 | 5596 | 5882289 |
| NA | 2019/04/27 | KP20194f | 5449011 | 194901 | 133772 | 87095 | 11970 | 5596 | 5882345 |

Chr: chromosome; p1-p5: plasmid 1 to plasmid 5

Supplementary Table 3. Single-nucleotide polymorphisms (SNPs) found in the 11 strains of the outbreak for which the complete genome sequence was obtained

| Position in KP20194a | KP20194a | KP20194a2 | KP20194b | KP20194b2 | KP20194c | KP20194c3 | KP20194c4 | KP20194c5 | KP20194d | KP20194e | KP20194f | Product | Effects on protein |
| --- | --- | --- | --- | --- | --- | --- | --- | --- | --- | --- | --- | --- | --- |
| 42101 | G | A | A | G | A | A | A | A | A | A | A | *ltnD* | V162A |
| 42136 | G | T | T | G | T | T | T | T | T | T | T | *ltnD* | Syn |
| 42307 | C | C | C | T | C | C | C | C | C | C | T | *ltnD* | Syn |
| 457163 | C | T | T | C | T | C | C | C | C | T | T | 23S rRNA | - |
| 1279558 | T | G | T | T | T | T | G | G | G | T | T | - | - |
| 2409845 | T | T | T | T | T | C | C | T | T | T | T | hypothetical | Y43H |
| 2805084 | A | G | G | G | G | G | G | G | G | G | G | *bla*_SHV-11_ | Syn |
| 3014925 | A | G | A | A | A | A | A | A | A | A | A | hypothetical | Syn |
| 3701729 | C | C | T | T | C | C | C | C | C | C | C | *GsiB* | R401G |
| 3720887 | G | G | G | G | G | A | G | G | G | G | G | *YbiT* | Syn |
| 4423094 | G | G | G | G | G | A | G | G | G | G | G | *ldcC* | Syn |
| 5252759 | G | G | G | G | G | A | G | G | G | G | G | *wzyE* | Syn |

Syn: synonymous mutation.

Supplementary Table 4. Strains used for phylogenetic tree analysis in this study

| Strain | Accession number | ST | Collection location | Collection date |  | SNPs difference | cgMLST difference |
| --- | --- | --- | --- | --- | --- | --- | --- |
| L79 | NLFC01000001.1 | 11 | China: Hangzhou | 2016/4/28 |  | 169 | 51 |
| ZWH183 | QMKB01000001.1 | 11 | China: Hangzhou | 2017 |  | 186 | 18 |
| L30 | NLDP01000001.1 | 11 | China: Hangzhou | 2016/3/10 |  | 159 | 15 |
| L39 | NLDZ01000001.1 | 11 | China: Hangzhou | 2016/3/16 |  | 159 | 15 |
| L513 | NLEM01000001.1 | 11 | China: Hangzhou | 2017/1/9 |  | 155 | 16 |
| L531 | NLEU01000001.1 | 11 | China: Hangzhou | 2017/1/20 |  | 158 | 18 |
| L20 | NLDG01000001.1 | 11 | China: Hangzhou | 2016/3/6 |  | 168 | 15 |
| L31 | NLDQ01000001.1 | 11 | China: Hangzhou | 2016/3/9 |  | 174 | 16 |
| L350 | NLDR01000001.1 | 11 | China: Hangzhou | 2016/10/31 |  | 200 | 13 |
| L529 | NLET01000001.1 | 11 | China: Hangzhou | 2017/1/20 |  | 169 | 40 |
| L256 | NLDM01000001.1 | 11 | China: Hangzhou | 2016/10/7 |  | 166 | 14 |
| L511 | NLEL01000001.1 | 11 | China: Hangzhou | 2017/1/10 |  | 171 | 17 |
| L223 | NLDK01000001.1 | 11 | China: Hangzhou | 2016/9/2 |  | 184 | 65 |
| SCKP040072 | PWAM01000001.1 | 11 | China: Sichuan | 2017/5/24 |  | 199 | 63 |
| XPY207 | NXNH01000001.1 | 11 | China: Hangzhou | 2016/9/19 |  | 195 | 35 |
| sklx33367 | NXEE01000001.1 | 11 | China: Hangzhou | 2015 |  | 155 | 27 |
| XPY20 | PCFS01000001.1 | 11 | China: Hangzhou | 2016/3/14 |  | 172 | 30 |
| K28 | QOVH01000001.1 | 11 | China: Zhejiang | 2020/10/17 |  | 101 | 23 |
| KP47432 | QURI01000001.1 | 11 | China: Hangzhou | 2017 |  | 66 | 36 |
| KP18-3-8 | CP048430.1 | 11 | China | 2018/7/14 |  | 88 | 23 |
| KP58 | CP041373 | 11 | China: Hangzhou | 2018/5/4 |  | 235 | 31 |
| BJCFK909 | CP034123.1 | 11 | China: Zhejiang | - |  | 121 | 27 |
| L39_2 | CP033954.1 | 11 | China: Zhejiang | 2018 |  | 117 | 15 |
| L388 | CP029220.1 | 11 | China: Zhejiang | 2017 |  | 195 | 36 |
| KP18-2079 | CP048933.1 | 11 |  | 2018/11/22 |  | 171 | 44 |
| SCKP020079 | CP029384.2 | 11 | China: Sichuan | 2016/12/6 |  | 143 | 31 |
| C789 | CP034415.1 | 11 | China: Shandong | 2016/4/14 |  | 128 | 33 |
| FDAARGOS_444 | CP023941.1 | 11 | Canada: BC | 2020/7/13 |  | 173 | 54 |
| Kp36 | CP047192.1 | 11 | China: Shanghai | 2019/1/10 |  | 169 | 39 |
| XJ-K2 | CP032240.1 | 11 | China: Shanghai | 2018/5/12 |  | 218 | 43 |
| WCHKP015093 | CP036300.1 | 11 | China: Chengdu | 2017/4/5 |  | 171 | 67 |
| WCHKP3 | CP031721.1 | 11 | China: Sichuan | 2020/9/16 |  | 163 | 68 |
| WCHKP115069 | CP033405.1 | 11 | China: Sichuan | 2018 |  | 225 | 92 |
| WCHKP649 | CP026585.3 | 11 | China: Sichuan | 2015/11/12 |  | 214 | 61 |
| WCHKP015625 | CP033396.1 | 11 | China: Sichuan | 2017 |  | 190 | 74 |
| WCHKP115068 | CP036365.1 | 11 | China: Sichuan | 2018 |  | 177 | 73 |
| WCHKP2080 | CP036361.1 | 11 | China: Chengdu | 2020/6/16 |  | 166 | 66 |
| WCHKP36 | CP028583.2 | 11 | China: Chengdu | 2017 |  | 178 | 68 |
| KP20194f | CP054720 | 11 | China: Loudi | 2019/4/27 |  | 5 | 0 |
| KP20194e | CP054726 | 11 | China: Loudi | 2019/4/25 |  | 4 | 0 |
| KP20194d | CP054732 | 11 | China: Loudi | 2019/4/24 |  | 4 | 0 |
| KP20194c5 | CP054738 | 11 | China: Loudi | 2019/4/15 |  | 4 | 0 |
| KP20194c4 | CP054744 | 11 | China: Loudi | 2019/4/15 |  | 5 | 0 |
| KP20194c3 | CP054750 | 11 | China: Loudi | 2019/4/15 |  | 7 | 3 |
| KP20194c | CP054756 | 11 | China: Loudi | 2019/4/13 |  | 4 | 0 |
| KP20194b2 | CP054762 | 11 | China: Loudi | 2019/4/12 |  | 3 | 1 |
| KP20194b | CP054768 | 11 | China: Loudi | 2019/4/3 |  | 5 | 1 |
| KP20194a2 | CP054774 | 11 | China: Loudi | 2019/4/12 |  | 5 | 0 |
| KP20194a | CP054780 | 11 | China: Loudi | 2019/4/3 |  | reference strain | reference strain |
