# Supplementary figures and images for: Co-occurrence of Rapid Gene Gain and Loss in an Interhospital Outbreak of Carbapenem-Resistant Hypervirulent ST11-K64 Klebsiella pneumoniae
Source: Front Microbiol. 2020 Nov 12;11:579618. doi: 10.3389/fmicb.2020.579618 (PMC7688671; doi:10.3389/fmicb.2020.579618)

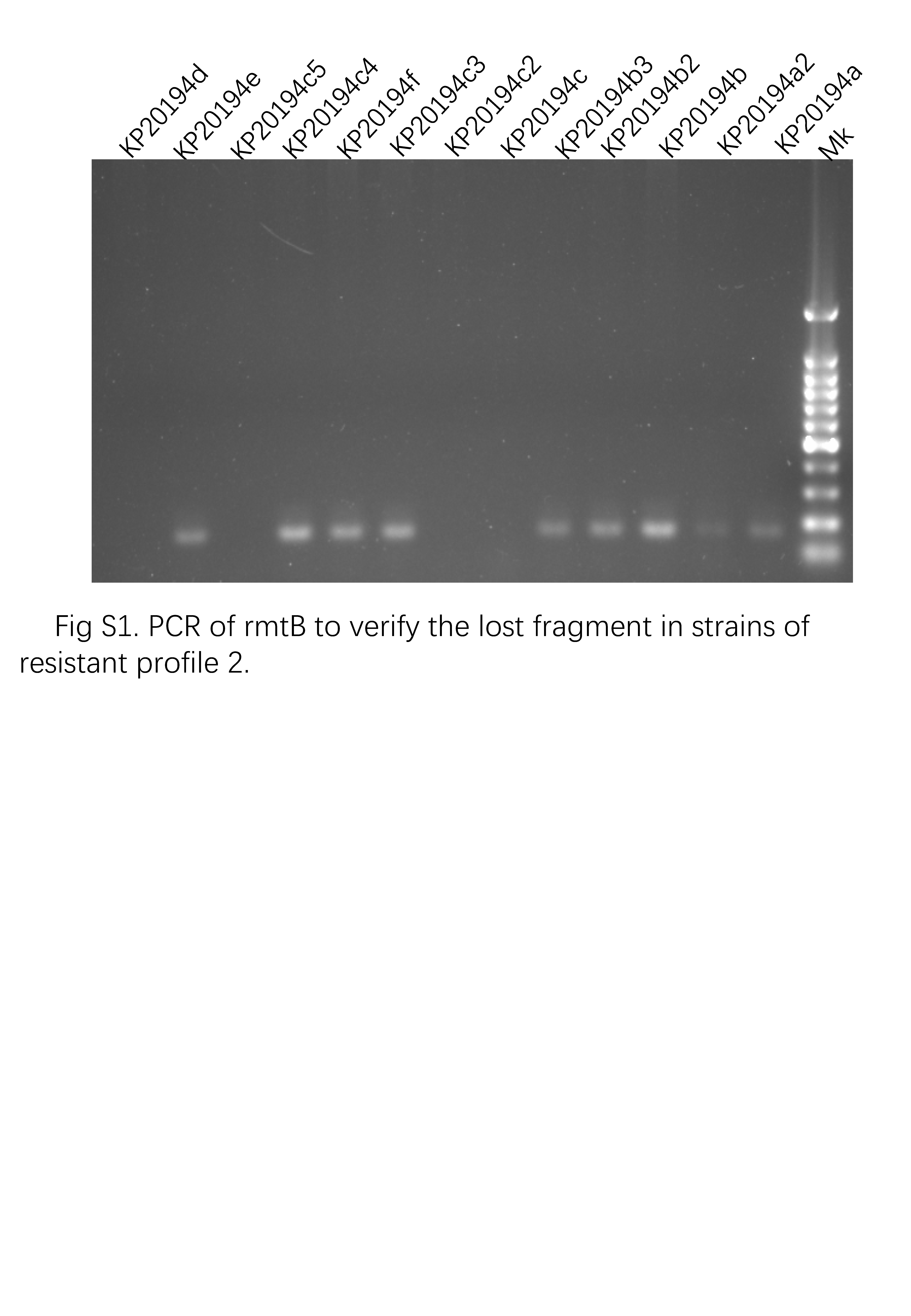

Supplement: Supplementary file 2 [file Image_1.TIF]

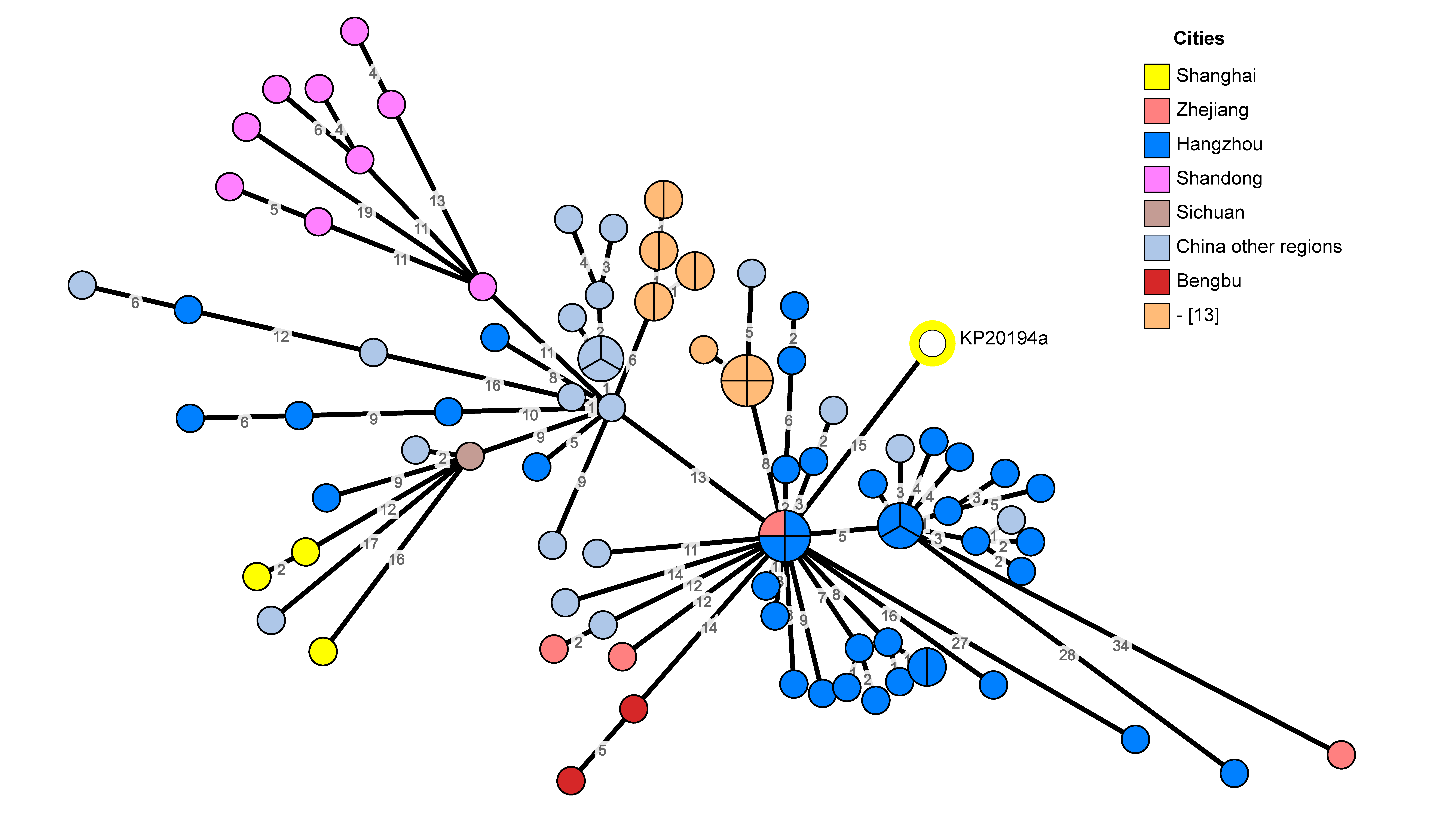

Supplement: Supplementary file 3 [file Image_2.TIFF]
